# How Does Government Information Service Quality Influence Public Environmental Awareness?

**DOI:** 10.3390/ijerph20010177

**Published:** 2022-12-22

**Authors:** Zhiwei Wang, Qiang Liu, Bo Hou

**Affiliations:** 1 School of Economics and Management, China University of Mining and Technology, Xuzhou 221008, China; 2 Academy of Humanities and Social Sciences, Jiangsu Normal University, Xuzhou 221116, China

**Keywords:** government information service quality, public environmental awareness, perception of environmental pollution, sense of political efficacy

## Abstract

The development of public environmental awareness has been an essential part of environmental governance in China and a prerequisite for the emergence of conscious environmental behaviors. However, the deeper factors influencing Chinese public environmental awareness are not yet fully understood. In this study, the perception of environmental pollution and the sense of political efficacy were introduced as variables to establish a public environmental awareness model, at the perception level, from the perspective of government information service quality. The correlations between different variables, as well as the working principles, were analyzed based on national environmental survey data. The results demonstrate that both external (the government information service quality) and psychological (the perception of environmental pollution and the sense of political efficacy) factors have significant positive influences on public environmental awareness, with the sense of political efficacy outperforming the perception of environmental pollution and the government information service quality. Analysis of the mediating effect reveals that government information service quality directly influences public environmental awareness; it also indirectly influences public environmental awareness through a chained mediating effect of the perception of environmental pollution and the sense of political efficacy. The results of the multiple-group analysis, with gender and urban–rural attributes as moderator variables, further show that, compared with men and urban residents, the impact of government information service quality and environmental pollution perception on women and rural people’s political efficacy is not significant. Therefore, it is suggested to optimize the government information service, improve the public’s perception of environmental pollution, and then promote the public’s sense of efficacy in the environment governance situation. Furthermore, the government should formulate differentiated strategies for different types of public to guide them to form a proactive awareness of environmental protection.

## 1. Introduction

With the rapid development of the natural sciences and production technology, the influence of human activities on the natural environment is incomparable to that which occurred before the industrial revolution. Rapid population and economic growth have been achieved at the expense of the over-consumption of resources and the huge discharge of environmental pollutants, resulting in various ecological issues caused by the decline in environmental quality globally. Constrained by the shortcomings of the traditional pollution prevention model, governments often choose to develop local economies at the cost of environmental pollution due to relaxing environmental regulations [1], resulting in poor environmental governance. According to the global environmental performance index (EPI) 2022 [2], China’s annual EPI score is just 28.4, ranking far below the average for countries at the same level of development. Recently, the Chinese government has been attaching great importance to environmental protection, and more and more Chinese citizens have begun to express their strong demand for the prevention of environmental pollution. As an under-developed section of environmental governance in China, the improvement of public environmental awareness would be of great importance in promoting green and low-carbon development and in providing solutions to ecological and environmental problems in China.

The concept of modern environmental awareness originated in western countries during the 1960s and 1970s as a reflection of the relationship between humans and nature [3]. Environmental awareness reflects public awareness of environmental issues and environmental protection. As an important sense of value, environmental consciousness reflects the public’s value orientation in relation to environmental issues, which is essentially the degree of importance attached to environmental protection [4]. Environmental governance practices in developed countries have revealed that government information service quality and the awakening of public environmental awareness are closely linked. Gupta [5] believed that administrative transparency is a fundamental of effective governance, and environment information services and public participation are essential for the global development of environmental governance. In response to the ecological crisis, major countries around the world are gradually transforming from a government-led environmental governance model to a collaborative environmental governance model in which the public is informed and involved in decision making. With the advance of service-oriented government, some local governments are trying to enhance public participation in comprehensive environmental pollution management by strengthening information disclosure. Although the optimization of government information services does not necessarily promote public participation in administrative decision making, the promotion of public participation in administrative decision making does require the optimization of government information services. This is because government information service quality is a prerequisite for public participation and support. Whether government information services, as an important tool for comprehensive environmental governance, can play a role in alleviating the situation of environmental pollution and in raising public environmental awareness in China is a topic of great research value.

During the modernization of the national governance systems, the improvement of public environmental awareness has gradually become an important indicator in the performance assessment of government departments. Previous studies have focused on macro- and mesoscopic analyses of public environmental awareness, including the construction of indicator systems [6] and surveys of environmental awareness among specific groups [7]. Additionally, the source [8] and presentation [9] of information services can affect the impacts of information, and the existing cognition or capability [10] and value preferences [11] of the public can affect their understanding of information. It has been demonstrated that information service quality is an important predisposing factor for public environmental awareness, but this neglects the influences of intermediate variables on public environmental awareness and pays insufficient attention to the influencing mechanism of psychology. As environmental awareness is an intrinsic factor that regulates, guides, and controls environmental behaviors, studying environmental awareness from the dimension of microscopic perception is necessary. Recent studies have revealed that the subjective perception of environmental pollution can influence public environmental concern [12] and that an increased sense of political efficacy would improve the public’s attitude toward environmental governance [13]. Therefore, this study uses a structural equation model to analyze the impact of government information service quality on public environmental awareness and further analyzes the relationship between influencing factors and their differences among groups, with the goal of providing scientific guidance for improving public environmental protection awareness and formulating relevant policies.

Throughout the existing research results, scholars have mainly carried out investigations and studies on specific groups from the macro level but have rarely measured or identified the factors affecting environmental awareness from the domain of public perception. They have also lacked in-depth discussion on the differences between groups of public environmental awareness. The differences between this study and previous studies are that environmental pollution perception and political efficacy variables are introduced to expand the model on the basis of the scientific analysis of public environmental awareness; the external constraint variables and psychological perception factors are included in the same analytical framework to build a theoretical model of public environmental awareness. Moreover, the individual characteristics of the public are selected as the moderating variable, and the differences in the moderating effects of the public environmental awareness are deeply compared through the method of grouping. Therefore, this study may provide a certain theoretical basis and policy reference for reducing the differences in the level of environmental awareness among different groups.

## 2. Theoretical Analysis and Hypotheses

### 2.1. Influences of Information Service Quality

To achieve its “peak carbon dioxide emissions” and “carbon neutrality” goals, the Chinese government has increasingly emphasized the important role that information services play in green transformation. The strengthening of information services by the government is in fact a process of empowering society and this new policy tool shows respect for the public’s right to access environmental information and strengthens the public’s oversight of polluting enterprises and local governments [14]. Peng et al. [15] investigated the foreign investment preferences of enterprises and found that improved environmental information service quality could enhance enterprises’ awareness of their environmental responsibility during the investment process. Zhang et al. [16] reported that information services are beneficial for addressing the issue of environmental pollution, while Tu et al. [17] also verified that environmental information services could play a significant role in environmental governance. From the perspective of the principal–agent relationship, information services can effectively alleviate information asymmetry between the government and the public, reduce public dissatisfaction with environmental issues, and thus reduce the public’s perception of environmental pollution. Therefore, Hypothesis 1 is proposed as follows.

**Hypothesis** **1.** *The quality of government environmental information service can significantly reduce the public perception of environmental pollution.*

Environmental information services are highly professional and information dissemination can help improve the public’s ability in relation to decision making about environmental governance; thus, the sense of political efficacy will increase accordingly [18]. Some researchers have proposed that exposure to news has a considerable impact on individuals’ sense of political efficacy [19]. The disclosure of environmental information can help eliminate public uncertainty and suspicion toward the government, enhance their expectations for the improvement of ecological and environmental issues, and let them believe that their political actions can influence environmental governance. Access to environmental information through government channels, especially participation in discussions on environmental governance, can enhance the public’s political knowledge, their ability to understand and use professional environmental information, and their sense of political efficacy. When information services are standardized, the quality of government regulation and services will be improved, environmental governance will enter a virtuous cycle [20], and the public will feel more motivated to tackle environmental challenges, as they believe they will be able to avoid the negative consequences of environmental governance.

As a top-down environmental policy, supplying information services has a positive effect on the public’s environmental awareness. A typical example is the 1962 book *Silent Spring* by Rachel Carson, which effectively triggered public awareness around environmental protection and promoted the rise of public environmental governance as a dominant force. The book filled an information gap in terms of the environmental quality and pollution detection measures taken by governments and greatly increased the public’s motivation to participate in environmental issues, although it was not an official report. Hence, information services are increasingly becoming an effective supplement to conventional environmental regulation measures, playing an irreplaceable role in increasing environmental awareness among the public and attracting public participation. As a result, the following hypothesis is proposed: a high-quality government environmental information service can lead to a significantly reduced public perception of environmental pollution and a significantly enhanced public sense of political efficacy and environmental awareness. Based on the above analysis, the following hypotheses are proposed:

**Hypothesis** **2.** *The quality of government environmental information service can significantly reduce the public perception of environmental pollution and significantly improves the public sense of political efficacy and environmental awareness.*

**Hypothesis** **3.** *The quality of government environmental information service can significantly improve the public environmental awareness.*

### 2.2. Influences of the Perception of Environmental Pollution

The perception of environmental pollution is a subjective judgment made by individuals about objective environmental pollution that results from the impact of objective environmental pollution on the inner experience of the individuals [21]. Environmental issues do not directly affect the behaviors of an individual but indirectly play a role by triggering certain psychological perceptions in the mind of the individual. Thus, great attention should be paid to the subjective perception of environmental pollution. If the public is aware of the seriousness of environmental issues, they will show a strong ability to politically control and influence these problems and such a particular political attitude can predict public participation in environmental governance. In other words, individuals with a high perception of environmental pollution tend to drive environmental issues onto the policy agenda, continuously strengthen their confidence in their ability to organize and implement political behaviors, and thus generate a sense of efficacy that exceeds behavioral expectations. When the public perceives the importance and urgency of environmental issues, they will naturally tend to pay more attention to environmental issues and the degree of this perception directly determines whether individuals can influence the political process [22].

The perception of environmental pollution is not only reflected in the attitude of political evaluation but also the sense of environmental responsibility and value preferences of individuals. A high level of environmental pollution in a region can increase the sensitivity of the local population to environmental issues and thus increase their environmental awareness. According to the pollution-driven model, the degradation of environmental quality affects the quality of life of the public and, thus, the public tends to pay more attention to local environmental issues. For example, exposure to pollutants may influence public environmental awareness more than social and cultural factors [23]. Additionally, public perception of the pollution situation can enhance social interactions between groups, i.e., the more seriously the residents perceive the environmental pollution situation to be, the more frequently the residents discuss it with their families, friends, and other nearby persons. Through social interactions, the environmental awareness of residents is often considerably enhanced [24]. Therefore, the following hypotheses are proposed:

**Hypothesis** **4.** *The perception of environmental pollution can significantly increase the public sense of political efficacy.*

**Hypothesis** **5.** *The perception of environmental pollution can significantly increase the public environmental awareness.*

### 2.3. Influences of the Sense of Political Efficacy

A sense of political efficacy is an attitudinal variable that affects public political participation; it is an individual’s judgment and an assessment of his or her ability to accomplish a political activity. It is a subjective perception of an individual’s political ability and the government’s response to the individual’s demands; it also reflects the likelihood of the public influencing political activities. Huang [25] conducted a study into the environmentally friendly behaviors of the public and found that individuals with higher self-efficacy tended to take a more responsible approach to environmental issues. Hong et al. [26] investigated the online learning condition of students during the COVID-19 pandemic and found that an increased sense of efficacy considerably increased learners’ perceptions of the usefulness of learning. Cai et al. [27] investigated the green behaviors of leaders of countries and found that a sense of efficacy plays a crucial role in fostering a sense of green innovation in teams. Milfont et al. [28] reported a study into the environmentally friendly attitudes of New Zealanders, which demonstrated the influence of a sense of political efficacy on public environmental awareness. As a result, Hypothesis 6 is proposed: a high sense of political efficacy leads to significantly enhanced public environmental awareness.

**Figure 1:**
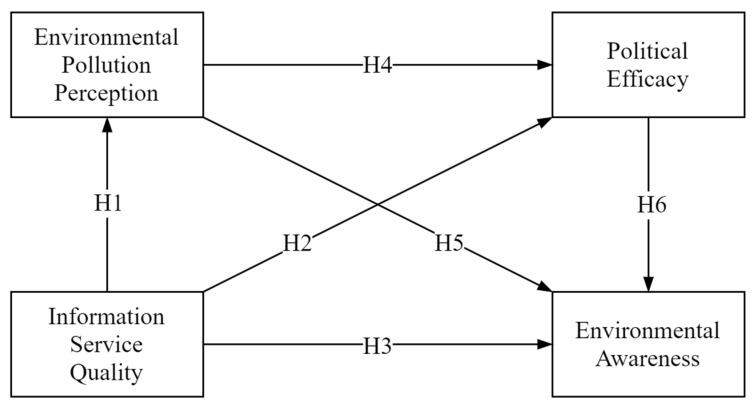
The influence mechanism of public environmental awareness.

Based on the above theories and hypotheses, this paper constructs the theoretical framework of the influence mechanism of public environmental awareness (Figure 1).

## 3. Research Design

### 3.1. Data Source and Sample Composition

The data selected for this study are derived from the Chinese Social Survey (CSS-2019) released by the Chinese Academy of Social Sciences in 2021. The Chinese Social Survey is a nationwide continuous sample survey project, covering 596 villages/neighborhood committees in 149 districts and counties in 31 provinces/autonomous regions/municipalities in China. The CSS-2019 interviewed more than 11,000 urban and rural households and collected 10,283 questionnaires. As the authoritative data source for studying social issues in China, the Chinese Social Survey provides authentic and comprehensive data for research on China in many fields and represents high academic research value. According to the needs of the study, the sections on government information service quality, perception of environmental pollution, sense of political efficacy, and public environmental awareness were selected, and 3686 valid cases were observed after the core variables were processed for missing data and outliers. The demographic characteristics of the sample are shown in Table 1.

### 3.2. Variable Selection

#### 3.2.1. Dependent Variables

Public environmental awareness is a complex multidimensional concept, for which the specific conceptual definition and measurement system have not yet been fully unified. Without considering specific environmental protection behaviors, this study followed the study design of Diekmann and Preisendoerfer [29] and divided environmental awareness into three dimensions: environmental knowledge, environmental value, and attitude to environmental protection. The corresponding questions in the CSS questionnaire were “I have neither knowledge nor capability in terms of environmental protection to comment”, “For China, economic development is more important than environmental protection”, and “It is the responsibility of the government to protect the environment, which has little to do with me”. The response options were “Very consistent”, “Consistent”, “Inconsistent”, and “Very inconsistent”. The larger the size of raw data, the higher the level of public environmental awareness.

#### 3.2.2. Independent Variables

The quality of the government information service is mainly represented by the degree of information disclosure and the service quality of public affairs provided by the government to the public. In the information system success model (DeLone and McLean IS success model), information quality and service quality are important predisposing factors influencing users’ willingness to continue using the information and the service [30]. Chien and Tsaur [31] found that information quality and service quality positively influence user satisfaction. The corresponding questions regarding information quality and service quality in the CSS questionnaire were “Government information is publicly available and government decisions and work are transparent” and “Have a sense of service and can respond to public demands in a timely manner”, which are intended to examine public satisfaction with government information services. The response options were “Very good”, “Good”, “Fair”, and “Bad”. In the present study, the values in the original questionnaire were reassigned in reverse order and the higher the processed value, the higher the quality of the government information service.

#### 3.2.3. Mediating Variables

The perception of environmental pollution is a negative inner experience in an individual’s mind, caused by objective environmental pollution. From the perspective of the public’s subjective perceptions, the environmental situation where they reside directly affects their daily lives and serves as a direct reference for them to assess the effectiveness of a government’s environmental governance program. Based on previous studies [32], we performed an assessment of three environmental factors: air quality, water quality, and noise. Respondents were asked “Are the following phenomena severe in your current residence”, which included references to air, water, and noise pollution. The response options were “Highly severe”, “Severe”, “Not so severe”, and “No such situation”. In this study, the values in the original questionnaire were reassigned in reverse order and the higher the processed value, the worse the public’s subjective perception of environmental pollution.

A sense of political efficacy is a subjective judgment of an individual toward the extent to which their political behaviors affect political activities; this can affect the value preferences and behavioral orientation of an individual. As a psychological perception factor, the sense of political efficacy can play a mediating role in influencing an individual’s understanding and perception of a certain thing [33]. It is usually divided into an intrinsic sense of efficacy and an extrinsic sense of efficacy. Specifically, the intrinsic sense of efficacy indicates that the public believes they are capable of political participation and can influence the government, while the extrinsic sense of efficacy indicates that the public believes the government can respond in a timely manner [34]. The corresponding statements in the CSS questionnaire were “I have both capability and knowledge to comment on politics”, “My freedom of speech will be restricted by the government”, and “It makes no difference for the public to participate in political activities as they can hardly have any fundamental influences on the government”. The response options were “Totally agree”, “Agree”, “Disagree”, and “Totally disagree”. In this study, the values in the original questionnaire were reassigned in reverse order and the higher the processed value, the greater the public’s sense of political efficacy.

There are 4 main variables in the study and the definitions and descriptive statistics of each variable are shown in Table 2. Overall, there was a relatively even distribution of scores across the respondents, with no significant differences between the higher and lower levels. Specifically, the public environmental awareness is at a medium level, with scores ranging from 2.63 to 3.16, and the score for environmental knowledge is higher than the score for environmental protection concept, which also shows that a certain stage of development is required from the mastery of environmental knowledge to the formation of environmental protection values, showing that the public has a certain degree of utilitarianism in dealing with the relationship between economic development and environmental protection. The scores for government information quality and government service quality are 2.86 and 2.82, respectively, indicating that most of the public generally agrees with the information disclosure and services provided by government departments. In the dimension of environmental pollution perception, air and water pollution scores are the least desirable, with more than 40% of respondents believing that the current air and water pollution is more serious. The sense of political efficacy score of no more than 3 indicates that the public has some perception and control of their participation in political life but it still needs to be strengthened.

### 3.3. Research Method

The proposed theoretical model has complex path relationships, and subjective errors were unavoidable in the measurement of latent variables. For this reason, structural equation modeling (SEM) was introduced for the empirical analysis. SEM consists of a measurement model that reflects the relationship between latent and measurable variables and a structural model that reflects the structural relationship between latent variables, represented by three matrix equations:

X = Λxξ + δ
(1)

Y = Λyη + ε
(2)

η = Bη + Γξ + ζ
(3)

Equations (1) and (2) are the measurement models, and Equation (3) is the structural model, where η and ξ are endogenous latent variable and exogenous latent variable matrices, respectively; Λx and Λy are the relationship coefficient matrices of observed variables X and Y, respectively; δ, ε, and ζ represent residual matrices; η is determined by B and the Γ coefficient matrix; and the error term ζ establishes a relationship between the endogenous latent variables and the exogenous latent variables to construct the SEM. This study specifically includes 4 latent variables and 11 observed variables.

## 4. Results and Analysis

### 4.1. Model Quality Test

#### 4.1.1. Common Method Bias Test

As the questionnaire was completed by self-reporting, the problem of common method bias among variables may exist. Hence, a Harman univariate test was conducted in this study. According to exploratory factor analysis, the variance explained by the first common factor was 18.709%, which was less than 40%, indicating that the findings were not affected by the homology problem. For the principal component analysis, a total of four common factors were extracted, with a cumulative variance contribution of 63.3%, which indicated that the variables could well reflect the raw data and that the analysis results were reliable.

#### 4.1.2. Reliability and Validity

In the exploratory factor analysis, four elements of the public environmental awareness model were extracted. However, the relationship between the elements and the factor loadings was unknown. For this reason, confirmatory factor analysis was conducted. As shown in Table 3, the standardized factor loadings of the four elements ranged from 0.566 to 0.927, which was greater than 0.5, indicating that these questions were effective in measuring the latent variables. Composite reliability (CR) reflects the consistency of all the questions among the latent variables and the higher the value, the higher the reliability of the questions. The CR values of the four elements ranged from 0.724 to 0.927, which was greater than 0.6, suggesting a high degree of internal consistency of the questions and stabilized measurement results. Average variance extracted (AVE) is an indicator of convergent validity among latent variables, and it reflects the ability of a set of questions to estimate a latent variable. The AVE values of the four factors ranged from 0.471 to 0.859, which met the judgment criterion of 0.4, suggesting a high validity among the factors and good operational definition.

#### 4.1.3. Goodness of Fit

The public environmental awareness model is a multidimensional abstract collection and only when the goodness of fit of SEM reaches a certain threshold can the path relationship be verified. The output results showed that RMSEA = 0.049 < 0.5, RMR = 0.029 < 0.08, PGFI = 0.565, and PNFI = 0.65, all of which were greater than 0.5, while CFI = 0.981, AGFI = 0.967, NFI = 0.953, and IFI = 0.958, all of which were greater than 0.9. All of these goodness-of-fit indices reached the ideal standard, indicating that the proposed model was acceptable and that hypothesis testing could be conducted.

### 4.2. Direct Path Analysis

The structural relationships between the latent variables and their standardized path coefficient estimates are shown in Table 4.

(1) The standardized path coefficient of government information service quality on public perception of environmental pollution is −0.230, which is significant at 99% confidence level. Hypothesis 1 is thus verified. Government information service quality and public perception of environmental pollution showed a significant negative relationship, indicating that the government’s efforts in disclosing environmental information and responding to the public’s demands had achieved the desired effect and thus the public’s negative experiences of environmental quality had been effectively alleviated. This study confirms that providing good information services can effectively improve the public’s perceptions of environmental pollution, which is consistent with the conclusions of Grimmelikhuijsen [35] and Peng et al., [36]. This suggests that the timely disclosure of air monitoring information to the public can increase the public’s environmental knowledge and improve the public’s perception of the government’s capabilities.

(2) The standardized path coefficients of government information service quality and environmental pollution perception on political efficacy are 0.091 and 0.080, respectively, which are significant at 99% confidence level. Hypotheses 2 and 4 are thus verified. Government information service quality showed a significant positive relationship with the public’s perception of environmental pollution and sense of political efficacy, suggesting that the higher the quality of the government information service, the more serious the public perceives environmental pollution to be and the higher their sense of political efficacy. This is highly consistent with previous work [37]. On the one hand, the information service serves as a reference for the public to influence government behaviors, with environment information disclosure enhancing the public’s oversight and accountability for government behaviors under the environmental governance. On the other hand, when the public is aware of the seriousness of environmental issues they will pay more attention to environmental policies, regulations, and policymaking projects, their ability to influence policy agenda setting will be strengthened, and thus their sense of political efficacy will be enhanced.

(3) The standardized path coefficients of government information service quality, environmental pollution perception, and political efficacy on public environmental awareness are 0.166, 0.055, and 0.433, respectively, and have passed the significance test at 95% confidence level. Thus, Hypotheses 3, 5, and 6 are supported by empirical results. Government information service quality, perception of environmental pollution, and sense of political efficacy showed a significant positive relationship with public environmental awareness, with the sense of political efficacy having the greatest effect. This is highly consistent with previous work [38] that the public’s belief that they can solve environmental issues has the most significant predictive effect on their environmental awareness. This can be attributed to the fact that the sense of efficacy is often seen as the psychological variable that is most closely associated with participation behaviors [39]. This reflects the public’s concern about environmental issues, perceptions, and likelihood of performing specific behaviors and interprets individuals’ political attitudes and feelings toward environmental governance. Considering that, this study further verified the indirect effects and effect sizes between the above-mentioned variables.

(4) Relationship between latent variables and measurable variables can be summarized as follows. Firstly, the impact of government service awareness (standardization path coefficient is 0.912) on the quality of government information service is greater than that of information disclosure provided by the government (standardization path coefficient is 0.789). A study in Seoul, South Korea also found supporting evidence that the quality of public services provided by the government contributed more to improving public evaluations [40]. Secondly, air pollution is the most significant factor in the public’s perception of environmental pollution. This is consistent with Yang’s research [41], which shows that the seriousness of air pollution is more important to the public than that of water pollution and noise pollution. Thirdly, the most significant factor in the latent variable of political efficacy is the degree to which people perceive freedom of speech. The study of online political participation environment by Lindsay et al., [42] also shows that the evaluation of the possibility of speech affecting the political system and its results is an important source for individuals to judge their ability to engage in political activities. Fourth, environmental knowledge, environmental values, and environmental protection attitudes can significantly affect public environmental awareness. The results are consistent with the research conclusion of Slavoljub et al., [43]. Improving the public’s education level, popularizing environmental knowledge, and strengthening the public’s sense of responsibility have positive policy implications for improving their environmental awareness and environmental behavior.

### 4.3. Mediating Effect

Given that the bias-corrected percentile bootstrap method significantly outperforms the conventional stepwise test for multiple mediating effects [44], in this study the mediating effects were examined by estimating the 95% bootstrap confidence interval. A total of 5000 bootstrap samples were randomly selected using the repeated sampling method; a confidence interval not including zero indicated a significant mediating path [45]. As shown in Table 5, the upper and lower bounds of the three paths did not include a zero, suggesting that the proposed chained mediating model is valid. Specifically, a sense of political efficacy showed the highest mediating effect, at 30%, suggesting that the quality of the government information service is very likely to indirectly affect public environmental awareness through the effect of the sense of political efficacy. Hence, promoting government information service quality, reducing the public’s perception of environmental pollution, and granting the public more rights to participate in social governance activities can considerably increase good perceptions of environmental governance among the public, thus attracting more people to participate in the improvement of the ecological environment.

### 4.4. Structural Equation Test of Subgroups

To further explore the moderating effects of gender and urban–rural attributes on public environmental awareness, this study introduces multiple-group SEM for path regression and then compares the differences in gender and urban–rural attributes on the impact of government information service quality on public environmental awareness. The path results for the subsamples are shown in Table 6.

In the SEM test, with gender as a moderating variable, the chi-square change of the model was 14.430, which showed a significant difference between the male and female samples in the structural model coefficients. The estimation results of the model show that the six pathways of the male group all pass the significance test, indicating that the chain mediating effect of the government information service quality on the male public environmental awareness is established. In the female subgroup, the two paths that the government information service quality affects sense of political efficacy (*P* = 0.486 > 0.05) and the perception of environmental pollution affects sense of political efficacy (*P* = 0.117 > 0.05) fail to pass the significance test, indicating that the government information service quality and perception of environmental pollution do not significantly promote the sense of the political efficacy of the female public. This finding is consistent with the research results of Arens and Watermann [46], showing that gender differences in the sense of political efficacy are significant. This may be related to women’s insensitivity to political life [47], suggesting a relative lack of awareness among women of their role in environmental protection. From the perspective of gender culture, both the Greek cultural tradition in the West and the Confucian cultural in China has weakened the role of women in political life. Female members are given more of a family caring role in the private sphere, often putting women at a disadvantage in terms of information services and environmental pollution in the public sphere.

In the SEM test of the urban–rural attribute as the moderating variable, the chi-square variation of the model is 25.049, which reaches the level of significance, indicating that there are significant differences between the urban and rural groups in the coefficient of the structural models. The estimation results of the model show that the six pathways of the urban group all pass the significance test, indicating that the chain mediating effect of government information service quality on the urban public environmental awareness is established. However, in the rural subgroup, the three paths fail to pass the significance test; that is, the government information service quality affects the sense of political efficacy (*P* = 0.869 > 0.05), the perception of environmental pollution affects the sense of political efficacy (*P* = 0.295 > 0.05), and the perception of environmental pollution affects environmental awareness (*P* = 0.776 > 0.05), indicating that government information service quality and the perception of environmental pollution do not significantly affect the sense of rural public political efficacy and the perception of environmental pollution significantly affects rural public environmental awareness. This is similar to the findings of Kaur et al., [48], where urban residents are more aware of the severity of environmental pollution and the harm to their health than rural residents. The possible reason is that air pollution, water pollution, and noise pollution in rural areas are not as serious as those in urban areas, so rural residents do not show a high level of environmental awareness.

## 5. Conclusions

The public is the beneficiary of ecological improvement and the undertaker of ecological problems. To improve China’s ecological environment, the internal motivation for the improvement of Chinese public environmental awareness should be explored. This paper added the perception of environmental pollution and the sense of political efficacy into the research framework of public environmental awareness, examined the influencing factors of public environmental awareness from the perspective of government information service, and examined whether there are differences in the influencing factors and degrees of public environmental awareness with different demographic characteristics based on gender and urban and rural attributes. Based on CSS data, this study investigated the mechanism of how government information service quality, perception of environmental pollution, and sense of political efficacy can influence public environmental awareness by means of structural equation modeling under the bootstrap independent sampling method. The results demonstrated that:

(1) Descriptive statistical analysis found that the public environmental awareness is at a medium level and there is still a lot of room for improvement from the mastery of environmental knowledge to the cultivation of environmental protection values. (2) In terms of the direct effects of SEM, government information services do improve the public’s perception of environmental pollution and increase the public’s sense of political efficacy and environmental participation. In terms of mediating effects, government information service quality contributes to the improvement of public environmental awareness through the chained mediating path of perception of environmental pollution and sense of political efficacy. Among them, the sense of political efficacy plays a significant promoting role. (3) Multiple-group analysis found that gender and urban–rural attributes have significant differences in the path of government information service quality affecting public environmental awareness. From the gender perspective, the chain mediating model that government information service quality affects male public environmental awareness is established, but the impact of government information service quality and environmental pollution perception on female public political efficacy is not significant. From the perspective of urban and rural regions, the chain mediating model that government information service quality affects the environmental awareness of urban residents is established, but the impact of environmental pollution perception on the environmental awareness of rural people is not significant.

The research conclusion has a positive reference value for the government to enhance the public’s environmental awareness. The degree of environmental information disclosure by government at all levels should be further increased to fully meet the public demand for environmental information. Moreover, the public’s environmental awareness and their evaluation of government could be improved and the construction of a modern environmental governance system could be further promoted. With regard to promoting environmental governance policies, it is not only necessary to publicize the information content of the government’s environmental governance work but also to publicize the harm caused by environmental pollution to the social environment and public life, so as to improve the public’s comprehensive understanding of the current environmental protection work. Additionally, the government should grant more rights to the public to make their voice heard in environmental governance, make them feel valued in environmental policymaking, make timely responses in environmental policy implementation, and shape the public’s perception of environmental governance to enhance the public’s intrinsic motivation for environmental protection. Particularly, the government should be more targeted in formulating relevant environmental governance policies and the public should be guided to participate in the construction of an environmental governance system according to local conditions. For example, for the urban public, we should focus on the important role of environmental pollution perception and form the positive feedback effect of environmental pollution perception on environmental awareness. With regard to the female public, the government can strengthen training to make them master effective methods of environmental protection and feel the importance of their participation in environmental practices, so as to improve their environmental protection awareness.

The multidimensional nature and complex characteristics of environmental awareness indicate that raising public awareness of environmental protection is an arduous social system project. Previous studies have confirmed the predictive effect of information services on public environmental awareness, but there have been limited studies into the underlying mechanism of how information services affect public environmental awareness. Therefore, from the perspective of government information service quality, this study introduced the perception of environmental pollution and the sense of political efficacy from psychological dimensions and investigated how these factors influence public environmental awareness and the degree of their influence. The contribution of this study lies in that we proposed the mediating effect transmission path of “information service quality—perception of environmental pollution—sense of political efficacy—environmental awareness” and we conducted an empirical analysis, which provides a valuable reference framework for subsequent studies. This can help further examine current environmental governance efforts in terms of government information services and public perceptions and thus promote coordinated environmental governance with the participation of multiple parties.

Nevertheless, this study exhibits several limitations. First, although the latest national survey data were used in this study, the cross-sectional design makes it hard to draw causal inferences and the hypothesized relationships between variables need to be further verified by follow-up surveys. Second, as the study of the correlation between government information services and public environmental awareness is still in its early stages, this study did not take into account sociocultural and personal background factors; the roles of subjective perceptions and social construction need to be further investigated.

## Figures and Tables

**Table 1:** Demographic characteristics of the sample.

Type	Option	Number	Frequency
Gender	Female	1971	53.5%
	Male	1715	46.5%
Region	Rural	1518	41.2%
	Urban	2168	58.8%
Age	18~44 years	1805	49.0%
	45~59 years	1261	34.2%
	≥60 years	620	16.8%
Education	Elementary school and below	911	24.7%
	Junior high school	1174	31.8%
	High school	755	20.5%
	Junior college	806	21.9%
	Undergraduate and above	40	1.1%

**Table 2:** The results of descriptive statistical analysis.

Variable	Variable Code	1	2	3	4	Mean	Std. Dev.
Public Environmental Awareness	Environmental knowledge	12.5%	23.5%	41.7%	22.2%	2.74	0.943
	Environmental value	15.3%	21.8%	47.6%	15.3%	2.63	0.920
	Environmental protection attitude	7.5%	10.2%	41.4%	40.9%	3.16	0.887
Government Information Service Quality	Information quality	5.6%	22.5%	51.7%	20.2%	2.86	0.796
	Service quality	6.5%	25.0%	48.9%	19.6%	2.82	0.820
Perception of Environmental Pollution	Air pollution perception	26.3%	48.1%	14.9%	10.8%	2.10	0.913
	Water pollution Perception	28.1%	43.2%	17.1%	11.6%	2.12	0.950
	Noise pollution perception	36.1%	40.5%	14.2%	9.2%	1.97	0.933
Sense of Political Efficacy	Believe in your ability	8.9%	38.2%	36.3%	16.6%	2.61	0.866
	Believe in your influence	16.7%	26.6%	42.5%	14.2%	2.54	0.931
	Believe you are unrestricted	10.6%	25.9%	49.5%	14.0%	2.67	0.845

**Table 3:** Reliability and validity analysis of measurement model.

Latent Variable	Observational Variable	Std.	AVE	CR
Information Service Quality (y_4_)	ISQ1 (x_10_)	0.927	0.859	0.924
	ISQ2 (x_11_)	0.927		
Environmental Pollution Perception (y_3_)	EPP1 (x_7_)	0.864	0.683	0.866
	EPP2 (x_8_)	0.820		
	EPP3 (x_9_)	0.794		
Political Efficacy (y_2_)	PE1 (x_4_)	0.566	0.471	0.724
	PE2 (x_5_)	0.803		
	PE3 (x_6_)	0.670		
Environmental Awareness (y_1_)	EA1 (x_1_)	0.801	0.566	0.796
	EA2 (x_2_)	0.740		
	EA3 (x_3_)	0.714		

**Table 4:** Regression results between SEM variables.

Path	Unstd.	S.E.	C.R.	P	Std.
ISQ→EPP	−0.222	0.020	−11.135	***	−0.230
ISQ→PE	0.037	0.011	3.322	***	0.091
EPP→PE	0.034	0.012	2.846	0.004	0.080
ISQ→EA	0.126	0.018	6.915	***	0.166
EPP→EA	0.044	0.019	2.292	0.022	0.055
PE→EA	0.815	0.089	9.112	***	0.433
ISQ→X_10_	1.000	—	—	—	0.789
ISQ→X_11_	1.191	0.072	16.448	***	0.912
EPP→X_9_	1.000	—	—	—	0.650
EPP→X_8_	1.107	0.034	32.598	***	0.708
EPP→X_7_	1.236	0.038	32.139		0.822
PE→X_4_	1.000	—	—	—	0.293
PE→X_5_	1.846	0.175	10.563	***	0.554
PE→X_6_	1.952	0.184	10.630	***	0.532
EA→X_1_	1.000	—	—	—	0.519
EA→X_2_	1.324	0.069	19.145	***	0.713
EA→X_3_	1.089	0.055	19.840	***	0.551

Note: *** denote significance level of 0.01.

**Table 5:** The results of the mediating effect test.

Path	Estimate	SE	Confidence Interval	*P*	Ratio
ISQ→EPP→EA	−0.01	0.005	[−0.02,−0.001]	0.027	10%
ISQ→PE→EA	0.03	0.012	[0.009,0.056]	0.005	30%
ISQ→EPP→PE→EA	0.006	0.003	[0.002,0.012]	0.008	6%
Total indirect effect	0.026	0.013	[0.004,0.054]	0.02	26%

**Table 6:** Heterogeneity analysis.

Path	Gender Differences		Urban–Rural Differences	
	Female	Male	Rural	Urban
ISQ→EPP	−0.249 ***	−0.232 ***	−0.200 ***	−0.258 ***
ISQ→PE	0.026	0.109 ***	−0.007	0.173 ***
ISQ→EA	0.156 *	0.167 ***	0.198 ***	0.143 ***
EPP→PE	0.061	0.086 ***	0.043	0.132 ***
EPP→EA	0.065 *	0.051 *	−0.011	0.057 *
PE→EA	0.489 ***	0.416 ***	0.436 ***	0.412 ***

Note: * and *** denote significance level of 0.10 and 0.01, respectively.

## Data Availability

Publicly available datasets were analyzed in this study. This data can be found here: http://sociology.cssn.cn/css_sy/zlysj/lnsj/.

## References

[1] Peng X. (2020). Strategic interaction of environmental regulation and green productivity growth in China: Green innovation or pollution refuge. Sci. Total Environ..

[2] Wolf M.J., Emerson J.W., Esty D.C., de Sherbinin A., Wendling Z.A. 2022 Environmental Performance Index. New Haven, CT: Yale Center Environmental Law & Policy.

[3] Maloney M.P., Ward M.P. (1973). Ecology: Let’s Hear from the People. An Objective Scale for the Measurement of Ecological Attitudes and Knowledge. Am. Psychol..

[4] Hu J.G., Pei Y. (2019). The Change of Public Environmental Awareness and Its Influencing Factors in China—Based on the Perspective of Post-materialism Theory. Acad. J. Jinyang.

[5] Gupta A. (2010). Transparency in global environmental governance: A coming of age. Global Environ. Polit..

[6] Alabi A.O. (2020). Assessment of Environmental Consciousness among Patrons in Selected Academic and Public Libraries in Lagos Metropolis. Electron. Green J..

[7] Zeng Y.X., Zhong L.S. (2017). Impact of tourist environmental awareness on environmental friendly behaviors: A case study from Qinghai Lake, China. J. Resour. Ecol..

[8] Hawkins D., Brook L.C., Hansen I.M., Hoopes N.A., Tidwell T.R. (2019). Do citizens see through transparency? Evidence from survey experiments in Peru. Brit. J. Polit. Sci..

[9] Piotrowski S., Grimmelikhuijsen S., Deat F. (2019). Numbers over narratives? How government message strategies affect citizens’ attitudes. Public Perform. Manag..

[10] Harrison E., Milton A.D., Richardson M.L. (2020). Knowledge and Perceptions of Environmental Issues by African Americans/Blacks in Washington, DC, USA: Giving Voice to the Voiceless. Sustainability.

[11] Yin S.J., Wang Y.Q., Li K. (2019). Pre-certification or retrospective?—The study of consumer preferences for food safety information labels and their interactive relationships. China Rural Sur..

[12] Ruan H., Qiu L., Chen J., Liu S., Ma Z. (2022). Government Trust, Environmental Pollution Perception, and Environmental Governance Satisfaction. Int. J. Environ. Res. Public Health.

[13] Boulianne S., Ohme J. (2022). Pathways to environmental activism in four countries: Social media, environmental concern, and political efficacy. J. Youth Stud..

[14] Kosajan V., Chang M., Xiong X., Feng Y., Wang S. (2018). The design and application of a government environmental information disclosure index in China. J. Cleaner Prod..

[15] Peng C., Fu W., Jiang H., Zou Y. (2022). The impact of environmental information disclosure on enterprises’ green preference of outbound investment: Evidence from China. Front. Psychol..

[16] Zhang S., Li Y., Hao Y., Zhang Y. (2018). Does public opinion affect air quality? Evidence based on the monthly data of 109 prefecture-level cities in China. Energy Policy.

[17] Tu Z., Hu T., Shen R. (2019). Evaluating public participation impact on environmental protection and ecological efficiency in China: Evidence from PITI disclosure. China Econ. Rev..

[18] Koo J., Kim J. (2016). The Effects of the SNS Activities of Politicians on Political Efficacy and the Intention to Participate in Voting. Asia Pac. J. Inf..

[19] Kim B., Barnidge M., Kim Y. (2019). The communicative processes of attempted political persuasion in social media environments: The mediating roles of cognitive elaboration and political orientations. Inform. Technol. Peopl..

[20] Chen M., Wang Y.Q., Yin S.J. (2019). Research on the Reformation Path of China’s Food Safety Certification Policy: From the Perspective of Consumer Preference.

[21] Li Y., Guan D., Tao S., Wang X., He K. (2018). A review of air pollution impact on subjective well-being: Survey versus visual psychophysics. J. Cleaner Prod..

[22] Levy B.L.M., Zint M.T. (2013). Toward fostering environmental political participation: Framing an agenda for environmental education research. Environ. Educ. Res..

[23] Mohai P. (1997). Gender differences in the perception of most important environmental problems. Race Gender Class.

[24] Hou J. (2020). Research on the Contractual Relationship of Contract Farming: Based on the Perspective of Behavioral Economics.

[25] Huang H. (2016). Media use, environmental beliefs, self-efficacy, and pro-environmental behavior. J. Bus. Res..

[26] Hong J.C., Liu X., Cao W., Tai K.H., Zhao L. (2022). Effects of self-efficacy and online learning mind states on learning ineffectiveness during the COVID-19 lockdown. Educ. Technol. Soc..

[27] Cai W., Yang C., Bossink B.A., Fu J. (2020). Linking leaders’ voluntary workplace green behavior and team green innovation: The mediation role of team green efficacy. Sustainability.

[28] Milfont T.L., Osborne D., Sibley C.G. (2022). Socio-political efficacy explains increase in New Zealanders’ pro-environmental attitudes due to COVID-19. J. Environ. Psycholo..

[29] Diekmann A., Preisendoerfer P. (2001). Umweltsoziologie: Eine Einführung.

[30] DeLone W.H., McLean E.R. (2003). The DeLone and McLean model of information systems success: A ten-year update. J. Manage. Inform. Syst..

[31] Chien S.W., Tsaur S.M. (2007). Investigating the success of ERP systems: Case studies in three Taiwanese high-tech industries. Comput. Ind..

[32] Brody S.D., Peck B.M., Highfield W.E. (2004). Examining localized patterns of air quality perception in Texas: A spatial and statistical analysis. Risk Anal..

[33] Ni J., Shen Y., Chen C., Liu X. (2022). The influence of occupational values on college students’ willingness to apply for civil servants: The mediating role of political efficacy. Front. Psychol..

[34] Acock A., Clarke H.D., Stewart M.C. (1985). A new model for old measures: A covariance structure analysis of political efficacy. J. Polit..

[35] Grimmelikhuijsen S. (2012). Linking transparency, knowledge and citizen trust in government: An experiment. Int. Rev. Adm. Sci..

[36] Peng M., Zhang H., Evans R.D., Zhong X., Yang K. (2019). Actual air pollution, environmental transparency, and the perception of air pollution in China. J. Environ. Dev..

[37] Ma L. (2017). Performance management and citizen satisfaction with the government: Evidence from Chinese municipalities. Public Admin..

[38] Roberts J.A. (1996). Green consumers in the 1990s: Profile and implications for advertising. J. Bus. Res..

[39] Easton D., Dennis J. (1967). The child’s acquisition of regime norms: Political efficacy. Am. Polit. Sci. Rev..

[40] Im T., Lee S.J. (2012). Does Management Performance Impact Citizen Satisfaction?. Am. Rev. Public Adm..

[41] Yang T. (2020). Association between perceived environmental pollution and health among urban and rural residents-a Chinese national study. BMC Public Health.

[42] Hoffman L.H., Jones P.E., Young D.G. (2013). Does my comment count? Perceptions of political participation in an online environment. Comput. Hum. Behav..

[43] Slavoljub J., Zivkovic L., Sladjana A., Dragica G., Zorica P.S. (2015). To the environmental responsibility among students through developing their environmental values. Procedia-Soc. Behav. Sci..

[44] Taylor A.B., MacKinnon D.P., Tein J.Y. (2008). Tests of the three-path mediated effect. Organ. Res. Methods.

[45] Zhao X., Lynch J.G., Chen Q. (2010). Reconsidering Baron and Kenny: Myths and truths about mediation analysis. J. Consum. Res..

[46] Aren A.K., Watermann R. (2017). Political efficacy in adolescence: Development, gender differences, and outcome relations. Dev. Psychol..

[47] Jennings M.K. (1998). Gender and political participation in the Chinese countryside. J. Polit..

[48] Kaur D., Sidhu M., Bal S., Sandhu P. (2015). Awareness of indoor pollution in rural and urban houses of Ludhiana district. Asian J. Home Sci..

